# Learning Burnout: Evaluating the Role of Social Support in Medical Students

**DOI:** 10.3389/fpsyg.2021.625506

**Published:** 2021-02-22

**Authors:** Jia-Yu Zhang, Tao Shu, Ming Xiang, Zhan-Chun Feng

**Affiliations:** ^1^School of Medicine and Health Management, Tongji Medical College, Huazhong University of Science and Technology, Wuhan, China; ^2^Medical Academic Affairs Office, Tongji Medical College, Huazhong University of Science and Technology, Wuhan, China

**Keywords:** learning burnout, social support, medical students, China, survey

## Abstract

**Background:** Burnout is a stress-induced syndrome considered to be closely related to work. Although social support could relief burnout syndrome, its effect on learning burnout in medical students remains unclear. The objectives of the study are to evaluate the association between learning burnout and social support in Chinese medical students.

**Methods:** A cross-sectional online survey was distributed to students who participated in online learning in a medical college in Wuhan during the COVID-19 epidemic. We used the Lian version of the Maslach Burnout Inventory (MBI) to assess learning burnout and the Social Support Rating Scale (SSRS) to assess social support. Chi-square tests were used to analyze factors associated with burnout. Independent *t*-test and multiple logistic regression were explored to analyze the relationship between social support and burnout.

**Results:** A total of 684 students have completed the survey (response rate: 30.9%), of which 315 (46.12%) met standard criteria for learning burnout. Multiple logistic regression analysis has revealed that seniors, low family income and low social support were significant predictors of learning burnout (χ^2^ = 41.983, *p* < 0.001). After adjusting for the grade and family income, there was a significant and relevant association between social support and learning burnout (OR = 0.937; 95% CI: 0.905–0.970; *p* < 0.001).

**Conclusions:** Learning burnout was highly prevalent in medical students at our college. Senior students and low family income might be risk factors for learning burnout. Social support, especially subjective support and utilization of support might play a protective role in reducing the risk of learning burnout.

## Practice Points

Social support, especially subjective support and utilization of support has a positive effect on alleviating learning burnout.

## Introduction

Burnout is a state of psychological distress, which is widely considered an important work-related syndrome. Research on burnout has been done on the basis of the sample of practicing physicians in medical fields. However, more and more attentions are recently paid to college, students among which could also be affected by burnout. The burnout is called learning burnout or academic burnout, which is originated from the definition of burnout and concludes the same core elements (Pagnin et al., 2013; Cheng et al., 2019). Learning burnout is defined as a combination of emotional exhaustion, cynicism and academic inefficacy due to their failure in meeting academic requirements (Schaufeli et al., 2002; Salmela-Aro et al., 2009).

Learning burnout has higher prevalence among medical students. A systematic review reported 25.8–52.1% of medical students had above the average level of burnout in China (Chunming et al., 2017). The wide range of burnout levels might be explained by the use of different definitions, measurements, and study designs. Moreover, learning burnout has serious negative effects on students and the quality of health care services. It could undermine medical students' professional development, diminish personal, and professional qualities (e.g., honesty, integrity, altruism, self-regulation) (Brazeau et al., 2010; Dyrbye et al., 2010; Paro et al., 2014), and cause problems such as increased medical errors, reduced quality of patient care, and low patient satisfaction (Fahrenkopf et al., 2008; Wallace et al., 2009). As a result, much more attention has been paid to learning burnout among medical students.

A lot of factors contribute to the risk of learning burnout, such as gender, grade, family economic aspects, employment status (Pan, 2011; Jiang, 2014). According to China Medical Students Survey 2019, 65% of clinical medical students' family income was <100,000 (CNY). Moreover, it generally takes more than 11 years for them to obtain a doctoral degree (bachelor degree: 5 years, master's degree: 3 years and doctor's degree: at least 3 years). Therefore, medical students may face more economic and academic pressure. Conversely, social support was regarded as a protective factor to reduce medical residents' risk of burnout (Huang et al., 2020). Social support is defined as the assistance and protection provided by others through formal or informal means (Cañadas-De la Fuente et al., 2015). The absence of social support was considered to be one of the main stress-inducing factors, a predictor of developing burnout (García-Izquierdo and Ríos-Rísquez, 2012). Good social support and other organizational factors such as good feedback and leadership were correlated with low levels of burnout in work environments (Melchior et al., 1997). Similarly, medical students with higher levels of social support were less likely to have burnout symptoms (Prins et al., 2007).

The COVID-19 broke out in Wuhan and quickly swept over China since January 2020. Our government promptly implemented nationwide public health emergency measures such as home isolation to control the spread of COVID-19. Besides, the government and schools have taken many measures to alleviate the impact of the epidemic on students, including providing living materials, increasing student subsidies, and psychological counseling. Therefore, social support was especially important during this period and has attracted much attentions in medical professionals (Gao et al., 2020; Luo and Huang, 2020). According to the China Education Statistics Yearbook of 2014 and literature report, there were about 200 medical colleges and universities with 1.1 million undergraduate medical students in China (Wu et al., 2020). A large number of students were still unable to return to school after the vacation, and most domestic universities including our school have adopted online teaching methods to maintain normal teaching arrangements.

Although psychological literature has confirmed that social support could reduce burnout through resilience (King et al., 1998; Amanda et al., 2012), it is still unknown whether it could alleviate learning burnout in medical students and its alleviation mechanism. In this study, we aimed (1) to investigate learning burnout of students in Tongji medical college in Wuhan after online learning for 2 months, and (2) to explore the relationship between social support and learning burnout, and (3) to seek the mechanism of social support to alleviate learning burnout according to social support theory, and (4) to determine the effect size of social support by controlling for several demographic predictors of learning burnout.

## Methods

### Data Collection

The target population consisted of medical students at the Tongji Medical College Huazhong University of Science and Technology. The electronic survey which was anonymous and confidential was distributed electronically by WeChat tools to 2,214 students who participated in online learning on April 2020 with the Wenjuanxing platform. Methods like logical verification, manual inspection, etc. were applied to ensure the quality of the survey. However, only 684 students completed the questionnaire due to some reasons, such as lack of Internet access, poor compliance, and so on. The study was approved by the institutional review boards of the Tongji Medical College Huazhong University of Science and Technology.

### Measures/Instruments

The self-administered questionnaire was divided into three parts.

#### Part 1 Demographic Characteristics

Part 1 consisted of demographic data, including age, gender, grade, residence, household income in 2019, whether to be a class leader during college, whether to receive a scholarship during college.

#### Part 2 Learning Burnout

We used the Learning Burnout Scale (LBS) to measure online learning burnout of undergraduate students. LBS was developed by Chinese researchers on the basis of MBI (Lian et al., 2005). It was made up of 20 items covering three domains of burnout: dejection (eight items), improper behavior (six items), and reduced personal accomplishment (six items). Items were scored on a 5-point Likert scale ranging from one (totally disagree) to five (totally agree). Additionally, the expression of some items was modified according to the characteristics of online learning (for example, changing the following “I felt exhausted after learning for a whole day” to “I felt exhausted after online learning for a whole day”). Learning burnout and its dimensions were measured in the form of the mean score and their cut-off points were three. The Cronbach's alpha achieved 0.907, 0.828, 0.787, and 0.776 in LBS and its dimensions of dejection, improper behavior, and reduced personal accomplishment, respectively.

#### Part 3 Social Support

Social support was assessed by the SSRS, which was developed by a Chinese researcher (Xiao, 1994). This widely utilized instrument includes three measurable dimensions of social support: subjective support (four items) and utilization of support (three items) and objective support (three items). Objective support reflects individuals' social networks and material direct support, and emotional support. Subjective support refers to perceptions of being respected, supported, and understood. Utilization of support reflects the extent to which respondents seek and make use of social support. Researchers usually made use of the total score to assess social support and its dimensions, and have demonstrated that SSRS had good predictive validity and internal consistency among Chinese medical students (Xiao, 1994; Zhang and Huang, 2007). Besides, we calculated the Cronbach's alpha (0.678) to measure the internal consistency of SSRS in the present study.

### Statistical Analysis

All analyses were performed with SPSS version 21 (IBM Corp., Armonk, NY, USA). Categorical variables were presented as numbers and percentages, and continuous variables as mean ± standard deviation. Categorical variables were compared with chi-square tests, and continuous variables with Student's *t*-tests or analysis of variance. Multivariate logistic regression analysis was conducted to evaluate the association between social support and learning burnout. A *p*-value of 0.05 (two-tailed) was considered to be statistically significant.

## Results

### Descriptive Statistics of Demographics and Learning Burnout

Out of 2,214 students who were invited to participate, 684 responses were received giving an overall response rate of 30.9%. Since medical students should study and live in the main campus in their first year, students in our college were mainly sophomores and juniors, accounting for 45.39 and 34.26%. The median age of the students was 20 years (range 17–24). Over fifty percent of respondents were female. The numbers (percentages) of municipalities or provincial capitals, prefecture-level cities, county-level cities, and town or rural areas were 132 (19.33%), 167 (24.45%), 199 (29.14%), and 185 (27.09%), respectively. More than three-fifths (63.69%) of the students' family income in 2019 bellows 100,000 (CNY). A total of 367 respondents served as class cadres and 388 respondents received scholarships during college. The results have showed that 315 (46.12%) students displayed evidence of learning burnout, with 370 (54.1%) reporting high dejection, 329 (48.1%) reporting high improper behavior, and 295 (43.19%) reporting high reduced personal accomplishment during the online study. Table 1 shows the demographic characteristics and learning burnout of the responding students.

**Table 1:** Demographic characteristics and learning burnout of respondents.

**Variables**	**Items**	***N* (%)**
Grade	Second	310 (45.39)
	Third	234 (34.26)
	Forth and above	139 (20.35)
Median age, years (range)		20 (17–24)
Gender	Male	290 (42.46)
	Female	393 (57.54)
Area of residence	Municipalities/provincial capitals	132 (19.33)
	Prefecture-level city	167 (24.45)
	County-level cities	199 (29.14)
	Town	65 (9.52)
	Rural areas	120 (17.57)
Family income in 2019 (CNY)	50,000 and bellow	197 (28.84)
	50,000–100,000	238 (34.85)
	100,000–150,000	111 (16.25)
	150,000–200,000	68 (9.96)
	200,000 and above	69 (10.1)
Whether to be a class leader during college	No	316 (46.27)
	Yes	367 (53.73)
Whether to obtain a scholarship during college	No	295 (43.19)
	Yes	388 (56.81)
Learning burnout	No	368 (53.88)
	Yes	315 (46.12)
Dejection	No	313 (45.83)
	Yes	370 (54.17)
Improper behavior	No	355 (51.98)
	Yes	328 (48.02)
Reduced personal accomplishment	No	383 (56.08)
	Yes	300 (43.92)

### Analysis of Differences in Variables According to Demographic Characteristics

Table 2 displays differences of the numbers and percentages of learning burnout and its subscales among demographic feature groups. We found a significant difference in rates of learning burnout between lower and higher graders (χ^2^ = 13.603, *p* < 0.01). Similarly, seniors showed more dejection and improper behavior and lower personal accomplishment (χ^2^ = 7.046, *p* < 0.05; χ^2^ = 14.333, *p* < 0.01; χ^2^ = 7.674, *p* < 0.05). However, differences in the prevalence of learning burnout, dejection, and reduced personal accomplishment between genders and age groups haven't been discovered, while the results indicated that the proportion of improper behavior increased with age (χ^2^ = 11.209, *p* < 0.05).

**Table 2:** Differences in learning burnout and its subscales by demographic characteristics.

**Variables**	**Learning burnout**		**Dejection**		**Improper behavior**		**Reduced personal accomplishment**	
	***N* (%)**	**χ**^**2**^ **(p)**	***N* (%)**	**χ**^**2**^ **(p)**	***N* (%)**	**χ**^**2**^ **(p)**	***N* (%)**	**χ**^**2**^ **(p)**
**Grade**								
Second	123 (39.68)	**13.603**	153 (49.35)	**7.046**	127 (40.97)	**14.333**	124 (40.00)	**7.674**
Third	111 (47.44)	**(0.001)**	130 (55.56)	**(0.030)**	118 (50.43)	**(0.001)**	101 (43.16)	**(0.022)**
Forth and above	81 (58.27)		87 (62.59)		83 (59.71)		75 (53.96)	
**Age**								
18 and bellow	4 (28.57)	6.661	7 (50)	0.666	3 (21.43)	**11.209**	6 (42.86)	4.602
19–20	163 (42.78)	(0.084)	202 (53.02)	(0.888)	172 (45.14)	**(0.011)**	154 (40.42)	(0.203)
21–22	132 (51.36)		144 (56.03)		132 (51.36)		124 (48.25)	
23 and above	16 (51.61)		17 (54.84)		21 (67.74)		16 (51.61)	
**Gender**								
Male	131 (45.17)	0.182	162 (55.86)	0.579	151 (52.07)	3.305	122 (42.07)	0.704
Female	184 (46.82)	(0.670)	208 (52.93)	(0.447)	177 (45.04)	(0.069)	178 (45.29)	(0.401)
**Residence**								
Municipalities/Provincial capitals	51 (38.64)	7.603	61 (46.21)	5.713	55 (41.67)	**9.624**	45 (34.09)	**14.336**
Prefecture-level city	77 (46.11)	(0.107)	94 (56.29)	(0.222)	84 (50.3)	**(0.047)**	69 (41.32)	**(0.006)**
County-level cities	91 (45.73)		110 (55.28)		89 (44.72)		85 (42.71)	
Town	29 (44.62)		33 (50.77)		29 (44.62)		35 (53.85)	
Rural areas	67 (55.83)		72 (60.00)		71 (59.17)		66 (55.00)	
**Family income in 2019 (CNY)**								
50,000 and bellow	110 (55.84)	**13.9**	119 (60.41)	7.4	109 (55.33)	**11.021**	108 (54.82)	**16.233**
50,000–100,000	110 (46.22)	**(0.008)**	131 (55.04)	(0.119)	119 (50.00)	**(0.026)**	102 (42.86)	**(0.003)**
100,000–150,000	41 (36.94)		57 (51.35)		43 (38.74)		42 (37.84)	
150,000–200,000	28 (41.18)		30 (44.12)		30 (44.12)		26 (38.24)	
200,000 and above	26 (37.68)		33 (47.83)		27 (39.13)		22 (31.88)	
**Whether to be a class leader during college**								
No	141 (44.62)	0.532	167 (52.85)	0.416	144 (45.57)	1.419	142 (44.94)	0.245
Yes	174 (47.41)	(0.466)	203 (55.31)	(0.519)	184 (50.14)	(0.234)	158 (43.05)	0.621
**Whether to obtain a scholarship during college**								
No	146 (49.49)	2.375	158 (53.56)	0.079	155 (52.54)	**4.248**	144 (48.81)	**5.041**
Yes	169 (43.56)	(0.123)	212 (54.64)	(0.779)	173 (44.59)	**(0.039)**	156 (40.21)	**(0.025)**

*If the statistical value in the variable is bolded, it means that the difference of the variable in this dimension is statistically significant*.

And no association was shown between students' residences and learning burnout (*p* = 0.107) and dejection (*p* = 0.222). But students whose family are located in economically developed cities like municipalities/provincial capitals had less improper behavior and lower personal accomplishment compared to the less developed areas (χ^2^ = 9.624, *p* < 0.05; χ^2^ = 14.336, *p* < 0.01). In contrast, the analysis of household income has indicated significant relationships with learning burnout. We also found 55.33 and 54.82% students with household income below 50,000 reported higher improper behavior (χ^2^ = 11.021, *p* < 0.05) and lower personal accomplishment (χ^2^ = 16.233, *p* < 0.05). Besides, the survey results have showed whether to be a class leader (*p* = 0.466) or to obtain a scholarship (*p* = 0.123) during college had no relationships with learning burnout. However, students having gained scholarships have presented with fewer symptoms of improper behavior and personal accomplishment than those who have not (χ^2^ = 4.248, *p* < 0.05; χ^2^ = 5.041, *p* < 0.05).

### Relationship Between Social Support and Learning Burnout

Student's *t*-tests were employed to identify if students' social support was affected by various learning burnout symptoms. Table 3 shows the relationship between social support and its different dimensions, and learning burnout and its related symptoms. Results have revealed that regardless of whether students presented learning burnout or not, social support was decreased if they exhibited the corresponding symptoms. Besides, a negative relationship was found between social support and learning burnout (*r* = −0.240; *p* < 0.01). Students, who exhibited a syndrome of learning burnout, had a lower score on subjective support and utilization of support (*t* = 4.510, *p* < 0.01; *t* = 4.158, *p* < 0.01**)**, but no difference in objective support (*t* = 1.128, *p* > 0.05**)**.

**Table 3:** Differences in students' SSRS and its dimensions' scores by learning burnout, dejection, improper behavior, and reduced personal accomplishment groups.

**Scale**	**Item**	**Social support**	***t***	**Subjective support**	***t***	**Objective support**	***t***	**Utilization of support**	***t***
			***p***		***p***		***p***		***p***
Learning burnout	Not burnout	27.86 ± 4.60	**4.370** ** <0.001**	12.14 ± 1.98	**4.510** ** <0.001**	8.29 ± 2.05	1.128 0.260	7.43 ± 2.12	**4.158** ** <0.001**
	Burnout	26.33 ± 4.53		11.44 ± 2.05		8.12 ± 1.93		6.77 ± 2.02	
Dejection	Not dejected	27.99 ± 4.52	**4.416** ** <0.001**	12.18 ± 1.94	**4.274** ** <0.001**	8.34 ± 2.00	1.506 0.133	7.48 ± 2.10	**4.116** ** <0.001**
	Dejected	26.45 ± 4.60		11.51 ± 2.08		8.11 ± 1.99		6.82 ± 2.05	
Improper behavior	Not have	27.93 ± 4.63	**4.602** ** <0.001**	12.21 ± 2.03	**5.393** ** <0.001**	8.28 ± 2.09	0.964 0.336	7.43 ± 2.12	**3.982** ** <0.001**
	Have	26.32 ± 4.48		11.39 ± 1.98		8.13 ± 1.89		6.80 ± 2.02	
Reduced personal accomplishment	Not competent	27.76 ± 4.49	**3.917** ** <0.001**	12.12 ± 1.94	**4.441** ** <0.001**	8.28 ± 2.06	1.048 0.295	7.36 ± 2.09	**3.310 0.001**
	Competent	26.38 ± 4.69		11.43 ± 2.11		8.12 ± 1.90		6.83 ± 2.07	

*If the statistical value in the variable is bolded, it means that the difference of the variable in this dimension is statistically significant*.

Multiple logistic regression was applied to identify the effect size of social support.

All the significant predictors of learning burnout in the univariate analysis were incorporated into the regression model, and the result was presented in Table 4. Grade, family income and social support have been found to show significant results in the final model (χ^2^ = 41.983, *p* < 0.001). After controlling for the two demographic factors, there was a significant and relevant association between social support and learning burnout (OR = 0.937; 95% CI: 0.905–0.970; *p* < 0.001) (Table 4).

**Table 4:** Multiple logistic regression of learning burnout.

**Factors**	**B**	**SE**	**Wald (**χ^2^)****	***p***	**OR (95% CI)**
**Grade**			12.952	0.002	
Second			-	-	1
Third	0.350	0.180	3.793	0.051	1.419 (0.998–2.019)
Forth and above	0.752	0.213	12.491	<0.001	2.120 (1.398–3.217)
**Family income in 2019 (CNY)**			10.222	0.037	
50,000 and bellow		-	-		1
50,000–100,000	−0.357	0.199	3.220	0.073	0.700 (0.474–1.033)
100,000–150,000	−0.719	0.250	8.287	0.004	0.487 (0.299–0.795)
150,000–200,000	−0.529	0.291	3.298	0.069	0.589 (0.333–1.043)
200,000 and above	−0.574	0.295	3.791	0.052	0.563 (0.316–1.004)
**Social support**	−0.065	0.018	13.715	<0.001	0.937 (0.905–0.970)
**Constant**	1.693	0.494	11.756	0.001	

## Discussion

### Main Findings

In this study, we examined the association between social support and learning burnout. It has been found that even after adjusting for the demographic factors, there was a significant and relevant association between the social support and learning burnout in the sample of Tongji medical college.

### Learning Burnout Prevalence

In this study, an average of 45.9% students has been found to have symptoms suggestive of learning burnout. The result was much higher than the rates of 21.76 and 36.46% of Chinese medical students, which were described by Tang et al. (2019) and Yang (2011), who used the same instrument and criteria. Students in Kingdom of Saudi Arabia had a moderate to high level of stress at the start of the COVID-19 outbreak (AlAteeq et al., 2020). Our students mainly came from Hubei province, which was the earliest and the worst area affected by COVID-19 in China. Therefore, they might have more physical and psychological stress, which was related to high burnout scores, during this period of home isolation.

### Demographic Factors Associated With Learning Burnout

In this study, it has been revealed that the prevalence of learning burnout and its dimensions was higher for students in more advanced years. The result was in line with previous research (Chunming et al., 2017), which might be due to the pressure of senior students facing employment or internships (Willcock et al., 2004; Dyrbye et al., 2006; Dahlin et al., 2007). Besides, the burnout rate of graduation students (63.6%) was much higher than that of non-graduation students (45.2%). Studies have also indicated that medical graduates faced more pressure, which involved in high burnout, than non-graduates (Iorga et al., 2018; Lal et al., 2020). The possible reason could be that the uncertainty about the pandemic effect might have increased their worry about graduating, finding a job or enrolling in further study (Tang et al., 2020).

It has been shown that environmental factors, especially those related to the economy, were closely related to learning burnout. Besides, whilst there was no significant difference in burnout rates of medical students from different residences or whether they were awarded scholarships, medical students from rural areas or without obtaining scholarships had a significantly higher rate in improper behavior or reduced personal accomplishment. The economic pressure might be considered to facilitate the development of burnout (Marisa et al., 2016; Manzano-García et al., 2017). Students with family difficulties or in rural areas had to face extra pressure of the economy from their families. This situation might give rise to more learning burnout.

### The Protective Effect of Social Support on Learning Burnout

Social support has a protective effect for burnout symptom in medical students (Kim et al., 2018). A similar effect of social support on learning burnout has been found, but the subjective support and utilization of support had a greater impact on learning burnout. A meta-analysis has also reported that seeking social support from friends or family members was already found to be correlated with burnout in a work setting (Halbesleben, 2006). Lazarus's stress and coping theory holds that active communication is an effective way to relieve stress. The results have also been demonstrated that subjective support and utilization of support could reduce learning burnout by communicating with relatives or friends. Social support provides a buffering effect against stress in that an individual who has more social support is also more resilient to stress (Cassidy, 1999). Besides, Thoits argued that social support served to regulate the stress itself and also provided a coping context, which could help the individual cope with stress or buffer the person against the demands (Thoits, 1986). This prompted us to make a further exploration on what social support environment and how other mechanisms of social support to alleviate learning burnout. Meanwhile, whether these mechanisms of social support could alleviate burnout in other population such as healthcare professionals may be worth to further research.

## Limitations

There are some limitations in the study at present. First, although the response rate of 30.9% was relatively low, a total of 684 samples have exceeded those of studies with higher response rates. Second, considering the differences in cultural context, measuring students' burnout with LBS instead of MBI might make up obstacles comparing with peers over the world. However, this scale had been widely used in China. Moreover, the sample drawn from a single school might be less generalizable to other schools and countries. Additionally, without investigation on the prevalence, the judgement whether the COVID-19 pandemic would indirectly affect students' learning burnout could not be made.

## Conclusion

The conclusion has been drawn that there was a high prevalence of learning burnout among students participating in online learning during the COVID-19 epidemic period. Students' grades and family income were all closely associated with learning burnout. Social support, especially subjective support and utilization of support might provide a protective effect against stress faced by medical students and hence decrease the possibility of their developing learning burnout. Considering the harmfulness of learning burnout, it is necessary to make a further look for other risk factors of learning burnout. Moreover, further research is also needed to identify other mechanisms of social support to relieve burnout in medical students or other population.

## Data Availability Statement

The original contributions presented in the study are included in the article/supplementary material, further inquiries can be directed to the corresponding authors.

## Ethics Statement

This study was carried out according to the ethical principles for medical research involving human subjects of the WMA Declaration of Helsinki. No individual data were collected, anonymity was guaranteed, participation was voluntary, and informed consent was obtained. The ethical board of Tongji Medical College determined that the study was exempt from formal ethical review.

## Author Contributions

J-YZ designed, analyzed and contributed in collecting the data, interpreting the results, and writing the draft manuscript. Z-CF contributed in guiding research design and revising the manuscript. TS and MX contributed in collecting and organizing the data. All authors read and approved the final manuscript.

## Conflict of Interest

The authors declare that the research was conducted in the absence of any commercial or financial relationships that could be construed as a potential conflict of interest.
